# Supplementary material for: Integration of genomics and transcriptomics predicts diabetic retinopathy susceptibility genes
Source: eLife. 2020 Nov 9;9:e59980. doi: 10.7554/eLife.59980 (PMC7728435; doi:10.7554/eLife.59980)
Supplement: Source code 1. [file elife-59980-code1.zip › SourceCode1.pdf]

```
library(dplyr)
library(limma)

# load data
dat<-local(get(load(file=paste(RData,"normalizedDataMatrix_filtered.RData",sep=""))))

probeList <- rownames(dat)  # probeID (nuID) gene annotation
if (require(lumiHumanAll.db) & require(annotate)){
  geneSymbol<- getSYMBOL(probeList, 'lumiHumanAll.db')
  geneName<- sapply(lookUp(probeList, 'lumiHumanAll.db', 'GENENAME'), function(x) x[1])
}
genes <-data.frame(ID=probeList, geneSymbol=geneSymbol, geneName=geneName,
stringsAsFactors=FALSE)

subject="all_replicate"
targets<-readTargets(paste(PhenotypeDir,"hg_sg_",subject,"_target.txt", sep=''))
Treat <- factor(targets$Treatment,levels=c("C","T"))
Replicates <- factor(targets$rep)
design <- model.matrix(~Replicates+Treat)

corfit <- duplicateCorrelation(dat, block = targets$Subject)
corfit$consensus.correlation
fit <-lmFit(dat,design,block=targets$Subject,correlation=corfit$consensus.correlation)
fit<-eBayes(fit)

qval.cutoff=0.1; FC.cutoff=0.17 # FC=1.12

x1=topTable(fit, coef="TreatT", n=nrow(genes),
p.value=qval.cutoff,adjust.method="BH",genelist=genes)
y1=topTable(fit, coef="TreatT", n=nrow(genes),adjust.method="BH",genelist=genes)

y2 = mutate(y1, sig=ifelse(y1$adj.P.Val<qval.cutoff & abs(y1$logFC) > FC.cutoff,"pval.cutoff",
"Not Sig"))

library(ggrepel)

vn=expression(paste("Response to glucose in all study subjects ", (RG[all])))
p <- ggplot(y2, aes(logFC, -log10(adj.P.Val))) +
  geom_point(aes(col=sig))  + theme(legend.position = "none", panel.grid.major =
element_blank(), panel.grid.minor = element_blank(),
                                  panel.background = element_blank(), axis.line = element_line(colour =
"black"))+
  scale_color_manual(values = c("gray", "red")) +
```

```
  ggtitle(vn) +
  xlab("log2FC")

p <- p + ggrepel::geom_text_repel(
  data = filter(y2, sig == "pval.cutoff"), aes(label = geneSymbol))

p
```
