# Supplementary material for: Integration of genomics and transcriptomics predicts diabetic retinopathy susceptibility genes
Source: eLife. 2020 Nov 9;9:e59980. doi: 10.7554/eLife.59980 (PMC7728435; doi:10.7554/eLife.59980)
Supplement: Source code 2. [file elife-59980-code2.zip › Sourcecode2.pdf]

```
## gsea up and down-regulated pathway QQplot

library(ggplot2)
library(gtools); library(bioDist); library(calibrate)
library(plyr); library(reshape2); library(scales)
library(ggfortify)
library(qqman)
library(data.table)

up<-fread("gsea_report_for_na_pos_1500567875906.xls")
down<-fread("gsea_report_for_na_neg_1500567875906.xls")


fdr2<-rbind(up,down)
fdr2_sorted<-fdr2[order(fdr2$`FDR q-val`),]
fdr2_sorted$FDR.q.val2<-fdr2_sorted$`FDR q-val`+1e-10
rm(down,fdr1)

qq(fdr2_sorted$FDR.q.val2, main = "RG_All", pch = 16, col=fdr1_sorted$group, cex = 0.8, las =
1, ylim=c(0,12))

legend('bottomright', legend = c('up-regulated', 'down-regulated'), pch = 16, col = c ("red",
"blue"), cex = 0.8)
```
