# Supplementary material for: Integration of genomics and transcriptomics predicts diabetic retinopathy susceptibility genes
Source: eLife. 2020 Nov 9;9:e59980. doi: 10.7554/eLife.59980 (PMC7728435; doi:10.7554/eLife.59980)
Supplement: Source code 3. [file elife-59980-code3.zip › Sourcecode3.pdf]

```
a=read.csv("/Users/ams/Downloads/All_treatment_effect_2017-06-13.csv", header=T)
hist(a$P.Value)
```
