# Supplementary material for: Integration of genomics and transcriptomics predicts diabetic retinopathy susceptibility genes
Source: eLife. 2020 Nov 9;9:e59980. doi: 10.7554/eLife.59980 (PMC7728435; doi:10.7554/eLife.59980)
Supplement: Source code 4. [file elife-59980-code4.zip › Sourcecode4.pdf]

```r
library(foreach)

library(doParallel)

library(ggplot2)

library(limma)

library(gtools); library(bioDist); library(calibrate)

library(plyr); library(reshape2); library(scales)

library(ggfortify)


HOME <- "/Grassi/Dexpr/"  # change if needed

InputDir <- paste(HOME,'data/input/', sep='')

InputFiles = list.files(path=InputDir, pattern='txt')

ControlDir <- paste(HOME,'data/control/', sep='')

ControlFiles = list.files(path=ControlDir, pattern='txt')

PhenotypeDir = paste(HOME,'phenotype/', sep='')

RData=paste(HOME,'rdata/',sep=''); dir.create(RData, showWarnings = FALSE)

Figs = paste(HOME,'Figs/', sep=''); dir.create(Figs, showWarnings = FALSE)

Output = paste(HOME,'output/', sep=''); dir.create(Figs, showWarnings = FALSE)


dat<-local(get(load(file=paste(RData,"normalizedDataMatrix_filtered.RData",sep=""))))

gene_annotation<-read.csv(paste(PhenotypeDir,"raw_delta_gene_anno.csv",sep=""))

rownames(gene_annotation)<-gene_annotation$GeneCode


# for normal samples

NoD<-dat[,grep("NoD",colnames(dat))] # grep all normal samples

DwC<-dat[,grep("DwC",colnames(dat))] # grep all DwC samples

DwoC<-dat[,grep("DwoC",colnames(dat))] # grep all DwoC samples


# separate high glucose and standard glucose

NoD_norm<-NoD[,grep("norm",colnames(NoD))]; NoD_30mM<-NoD[,grep("30mM",colnames(NoD))]
```

```
NoD_DE<-NoD_30mM-NoD_norm; colnames(NoD_DE)<-gsub("_30mM","",colnames(NoD_DE))


DwC_norm<-DwC[,grep("norm",colnames(DwC))]; DwC_30mM<-DwC[,grep("30mM",colnames(DwC))]

DwC_DE<-DwC_30mM-DwC_norm; colnames(DwC_DE)<-gsub("_30mM","",colnames(DwC_DE))


DwoC_norm<-DwoC[,grep("norm",colnames(DwoC))]; DwoC_30mM<-
DwoC[,grep("30mM",colnames(DwoC))]

DwoC_DE<-DwoC_30mM-DwoC_norm; colnames(DwoC_DE)<-gsub("_30mM","",colnames(DwoC_DE))


sg<-dat[,grep("norm",colnames(dat))]

hg<-dat[,grep("30mM",colnames(dat))]

All_DE<-hg-sg; colnames(All_DE)<-gsub("_30mM","",colnames(All_DE))

Diabetes<-cbind(DwC_DE,DwoC_DE)


DwC_hg_output<-var_proc(DwC_30mM,gene_annotation)

write.csv(DwC_hg_output, paste(Output,"hg_DwC_variation_",date,".csv",sep=""))

DwC_hg_output[,2:ncol(DwC_hg_output)]<-
data.frame(apply(DwC_hg_output[,2:ncol(DwC_hg_output)],2,as.numeric));


inter<-log2(DwC_hg_output$`inter-individual variance`)

intra<-log2(DwC_hg_output$`intra-individual variance`)

data<-as.data.frame(cbind(inter,intra))


#Fig 1 supplement 4

setEPS()

postscript(file = paste(Figs,"hg_DwC_inter_vs_intra-individual_density_",date,".eps",sep=""))

plot.multi.dens( list(inter, intra))

legend("topright", c("inter-individual","intra-individual"),col = c("black", "red"), lty = c(1, 1))

title(main="Proliferative diabetic retinopathy  (high glucose)")
```

```
dev.off()

var.test(b$inter.individual.variance, b$intra.individual.variance, alternative = "two.sided")

#Fig 1 supplement 3
ggd.qqplot = function(pvector, main=NULL, ...) {
  o = -log10(sort(pvector,decreasing=F))
  e = -log10( 1:length(o)/length(o) )
  plot(e,o,pch=19,cex=1, main=main, ...,
    xlab=expression(Expected~~-log[10](italic(p))),
    ylab=expression(Observed~~-log[10](italic(p))),
    xlim=c(0,max(e)), ylim=c(0,max(o)))
  lines(e,e,col="red")
}

NoD_sg_output<-var_proc(NoD_norm,gene_annotation)
p<-ggd.qqplot(NoD_sg_output$`p-value`,"No diabetes (standard glucose)")
p

#Fig 1 supplement 2
#------normal samples with standard glucose (sg)
NoD_sg_output<-var_proc(NoD_norm,gene_annotation)
write.csv(NoD_sg_output, paste(Output,"sg_NoD_variation_",date,".csv",sep=""))
NoD_sg_output[,2:ncol(NoD_sg_output)]<-
data.frame(apply(NoD_sg_output[,2:ncol(NoD_sg_output)],2,as.numeric));

inter<-log2(NoD_sg_output$`inter-individual variance`)
intra<-log2(NoD_sg_output$`intra-individual variance`)
data<-as.data.frame(cbind(inter,intra))
```

```
tmp<-cbind(log2(NoD_sg_output$`inter-individual variance`),log2(NoD_sg_output$`intra-individual variance`))

rownames(tmp)<-rownames(NoD_sg_output); colnames(tmp)<-c('inter-individual variance', 'intra-individual variance')


p<-ggplot(tmp, aes(y = `inter-individual variance`, x = `intra-individual variance`)) +
  geom_point(shape=1,size=0.1) +theme(text = element_text(size=20)) + theme(plot.title = element_text(hjust = 0.5)) +
  xlim(-10, 7.5) + ylim(-10, 7.5)+geom_abline(intercept=0, slope=1)+xlab("log2(intra-individual variance)") +
  ylab("log2(inter-individual variance)") + ggtitle("No diabetes (standard glucose)")
p
```
