# Supplementary material for: Integration of genomics and transcriptomics predicts diabetic retinopathy susceptibility genes
Source: eLife. 2020 Nov 9;9:e59980. doi: 10.7554/eLife.59980 (PMC7728435; doi:10.7554/eLife.59980)
Supplement: Source code 5. [file elife-59980-code5.zip › Sourcecode5.pdf]

```
library(dplyr)
library(limma)

# load data
dat<-local(get(load(file=paste(RData,"normalizedDataMatrix_filtered.RData",sep=""))))

probeList <- rownames(dat)  # probeID (nuID) gene annotation
if (require(lumiHumanAll.db) & require(annotate)){
  geneSymbol<- getSYMBOL(probeList, 'lumiHumanAll.db')
  geneName<- sapply(lookUp(probeList, 'lumiHumanAll.db', 'GENENAME'), function(x) x[1])
}
genes <-data.frame(ID=probeList, geneSymbol=geneSymbol, geneName=geneName,
stringsAsFactors=FALSE)

# PCA by group (HG, SG, delta)
# 1. No_DM
subject<-"No_DM"
NoD<-dat[,grep("NoD",colnames(dat))] # grep all normal samples
NoD_sg<-NoD[,grep("norm",colnames(NoD))]; NoD_hg<-NoD[,grep("30mM",colnames(NoD))];
NoD_delta <- NoD_hg-NoD_sg

# 2. No_PDR
subject<-"No_PDR"
DwoC<-dat[,grep("DwoC",colnames(dat))] # grep all normal samples
DwoC_sg<-DwoC[,grep("norm",colnames(DwoC))];
DwoC_hg<-DwoC[,grep("30mM",colnames(DwoC))];
DwoC_delta <- DwoC_hg-DwoC_sg

# 3. PDR
DwC<-dat[,grep("DwC",colnames(dat))] # grep all normal samples
DwC_sg<-DwC[,grep("norm",colnames(DwC))];
DwC_hg<-DwC[,grep("30mM",colnames(DwC))];
DwC_delta <- DwC_hg-DwC_sg

#1a. delta
delta<-cbind(DwC_delta,DwoC_delta,NoD_delta)
colnames(delta)<-substr(colnames(delta), 1, nchar(colnames(delta))-7)
subject="all_replicate"
targets<-readTargets(paste(PhenotypeDir,"delta_",subject,"_target.txt", sep=''))
group <- factor(targets$Group)

#The 0+ notation is telling R to not add an “intercept” column
# +group  ignoring any change due to group
```

```
#  interested in knowing genes related?
design <- model.matrix(~0+group)
colnames(design)<-levels(group)
rownames(design)<-targets$SampleName
corfit <- duplicateCorrelation(delta, block = targets$Subject)
fit <- lmFit(delta, design, block = targets$Subject, cor = corfit$consensus.correlation)

cm <- makeContrasts( delta_NoD_DwoC=NoD - DwoC, delta_NoD_DwC=NoD - DwC,
                     delta_NoD_Diabetes=NoD - (DwC+DwoC)/2, delta_DwC_DwoC = DwC-DwoC,
                     delta_DwoC_DwC = DwoC-DwC,
                     levels=design) # create a contrast matrix
fit2=contrasts.fit(fit,cm)
fit2=eBayes(fit2)

#---------------------
# delta DwC vs DwoC
#---------------------
pval.cutoff=0.05; FC.cutoff=0.263  #FC=1.2
x1=topTable(fit2, coef="delta_DwC_DwoC", n=nrow(genes),
adjust.method="BH",genelist=genes)
y1=topTable(fit2, coef="delta_DwC_DwoC",
n=nrow(genes),adjust.method="BH",genelist=genes)
x1_subset<-x1[x1$P.Value<pval.cutoff & abs(x1$logFC) > FC.cutoff,]
delta_DwC_DwoC_pval_FC<-x1_subset
s1=dplyr::select(delta_DwC_DwoC_pval_FC,geneSymbol,logFC,t, P.Value,adj.P.Val,B)

y2 = mutate(y1, sig=ifelse(y1$P.Value<pval.cutoff & abs(y1$logFC) > FC.cutoff,"pval.cutoff",
"Not Sig"))

vn=expression(paste("Response to glucose PDR vs. nDR ", (RG[all])))
p <- ggplot(y2, aes(logFC, -log10(P.Value))) +
  geom_point(aes(col=sig))  + theme(legend.position = "none", plot.title = element_text(hjust =
0.5), panel.grid.major = element_blank(), panel.grid.minor = element_blank(),
                                    panel.background = element_blank(), axis.line = element_line(colour =
"black"))+
  scale_color_manual(values = c("gray", "red")) +
  ggtitle(vn) +
  xlab("log2FC")

p <- p + ggrepel::geom_text_repel(
  data = filter(y2, P.Value<pval.cutoff  & abs(logFC) > FC.cutoff), aes(label = geneSymbol))+
xlim(-0.5,0.5)
```

p
