# Supplementary material for: Integration of genomics and transcriptomics predicts diabetic retinopathy susceptibility genes
Source: eLife. 2020 Nov 9;9:e59980. doi: 10.7554/eLife.59980 (PMC7728435; doi:10.7554/eLife.59980)
Supplement: Source code 6. [file elife-59980-code6.zip › Sourcecode6.pdf]

```
cd /projects/com_grassim/anamaria/anamaria/anamaria/hierar

module load compilers/r-3.6.1

library(data.table)
library(dplyr)
library(limma)
library(edgeR)
library(qvalue)
library(dplyr)
library(tibble)
library(ggpubr)
library(gplots)

##just to make file   ##### use this one!
a=fread("delta_DwC_DwoC_pvalue_0.05_log2FC_0.263_2017-09-13.csv", header=T)
al=select(a,ID,geneSymbol)
b=fread("delta_DwoC_DwC_pvalue_all_2017-11-23.csv", header=T)
bb=b[1:500,]
bl=select(bb,ID,geneSymbol)
t=rbind(al,bl)
#remove duplicates
tt=t[!duplicated(t), ]
#got 502 genes
write.table(tt, file="genes_toextract_502RG",  sep = " ", row.names = FALSE, col.names =
TRUE,quote=FALSE)
# just to make file end

get.data <- function(tissue, DataDir){

   file <- paste0(DataDir, tissue, ".RDS")
   data <- readRDS(file)

   txt <- paste0("model.matrix(~SEX + SMTSISCH + SMRIN + AGE +",
               paste(names(data$samples)[grep("SV", names(data$samples))], collapse="+"),
               ", data$samples)")
   design <- eval(parse(text = txt))

   logCPM <- cpm(data, log=TRUE, prior.count=3)

   design.nosvs <- model.matrix(~SEX + SMTSISCH + SMRIN + AGE, data$samples)

   return(list(design = design, logCPM = logCPM, count = data, design.nosvs = design.nosvs))
```

```
}


data=readRDS("Cells_EBV-transformed_lymphocytes.RDS")
count <- data[[1]]
logCPM <- data[[3]]
design <- data[[2]]
design <- design[, -c(1:7)]

v <- voom(count, design, plot=F)
y <- removeBatchEffect(v, covariate=design)

c=data[[3]]
cc=select(c,symbol)
yy=cbind(y,gene=cc$symbol)
d=as.data.frame(yy)
dd=noquote(d)

gg=read.table("ILL_gene", header=T)
ii=merge(x=dd,y=gg, by.x="gene", by.y="geneSymbol")
df = ii[!duplicated(ii$illumina_probe_id),]
rownames(df)=df$illumina_probe_id
df$illumina_probe_id<-NULL
df$gene<-NULL
dt=t(df)
tL=as.data.frame(dt)
df1 <- apply(tL, 2, function(x) as.numeric(as.character(x)))
rownames(df1) <- rownames(tL)
df1=as.data.frame(df1)

# for our Expression data
df2=read.table("all_inc",header=T)


res <- df1 %>% rownames_to_column('rnames') %>%
          bind_rows(df2 %>% rownames_to_column('rnames')) %>%
          column_to_rownames('rnames')

r=res[ , colSums(is.na(res)) == 0]

a <- t(apply(r, 1, function(x) qnorm(1-(rank(x)+1)/(length(x)+2))))
at=t(a)
```

```
genes=rownames(at)
aa=cbind(at,genes)
aat=noquote(aa)
aat=as.data.frame(aa)

#extract genes

g=read.table("genes_toextract_502RG", header=T)

gg=read.table("ILL_gene", header=T)

ii=merge(x=g,y=gg, by.x="geneSymbol", by.y="geneSymbol")
id=ii[!duplicated(ii$illumina_probe_id), ]

tot=merge(x=aat,y=id, by.x="genes", by.y="illumina_probe_id")
m=merge(x=tot,y=id, by.x="ID", by.y="ID")
m$genes<-NULL
m$geneSymbol<-NULL
m$ID<-NULL
mm=m[!duplicated(m$illumina_probe_id), ]
rownames(mm)=mm$illumina_probe_id
mm$illumina_probe_id<-NULL
d=t(mm)
dd=as.data.frame(d)


write.table(dd, file="dataforMDSplotRG_new",  sep = " ", row.names = TRUE, col.names =
TRUE,quote=FALSE)


#create MDS plot

library(data.table)
library(dplyr)
library(limma)
library(edgeR)
library(qvalue)
library(dplyr)
library(tibble)
library(ggpubr)
library(gplots)

a=read.table("/Users/ams/Desktop/dataforMDSplotRG_new", header=T)
```

```
a=a[175:218,]
at=t(a)
aN=at[ , grepl( "norm" , colnames(at) ) ]
a30=at[ , grepl( "30mM" , colnames(at) ) ]
arN <- t(apply(aN, 2, function(x) qnorm(1-(rank(x)+1)/(length(x)+2))))
ar30 <- t(apply(a30, 2, function(x) qnorm(1-(rank(x)+1)/(length(x)+2))))

#take the difference between ar_standard and ar_high
ar_diff = ar30-arN

#### for PDR and nPDR

ar_diff=ar_diff[1:15,]
mds <- (ar_diff) %>% dist() %>% cmdscale(k=2) %>% as_tibble()
mds$cols <- as.factor(c(rep("nPDR.rg",7),  rep("PDR.rg",8)) )


pdf(file = "RG.pdf")


for (dim1 in 1:2){
  for (dim2 in (dim1):2){
    d1 = paste0("Dim.",dim1); d2 = paste0("Dim.",dim2)
    colnames(mds)[c(dim1,dim2)] <- c(d1,d2)
    print(colnames(mds))
    print(ggscatter(mds, x = d1, y = d2 ,color ="cols", size=2 ,palette=c("blue","red")))
  }
}
dev.off()


# calculate p value
t.test(Dim.1 ~ cols, data = mds)


###RESULT

        Welch Two Sample t-test

data:  Dim.1 by cols
t = -8, df = 10, p-value = 3e-06
alternative hypothesis: true difference in means is not equal to 0
95 percent confidence interval:
```

-8.21 -4.66
sample estimates:

mean in group nPDR.rg	mean in group PDR.rg
-3.43	3.00
