# Supplementary material for: Integration of genomics and transcriptomics predicts diabetic retinopathy susceptibility genes
Source: eLife. 2020 Nov 9;9:e59980. doi: 10.7554/eLife.59980 (PMC7728435; doi:10.7554/eLife.59980)
Supplement: Source code 7. [file elife-59980-code7.zip › Sourcecode7.pdf]

```
# my QQ plot
qqunif = function(p, BH=T, MAIN = " ", SUB=" ")
{
  nn = length(p)
  xx =  -log10((1:nn)/(nn+1))
  plot( xx,  -sort(log10(p)),
       main = MAIN, sub= SUB, cex.sub=1.3,
       xlab=expression(Expected~~-log[10](italic(p))),
       ylab=expression(Observed~~-log[10](italic(p))),
       cex.lab=1.0,mgp=c(2,1,0))
  abline(0,1,col='red')
  if(BH) ## BH = include Benjamini Hochberg FDR
  {

    abline(-log10(0.05),1, col='black',lty=1)
    text(0.5,1.9 , "FDR=0.05", col = "gray60",srt=35, cex=1)
    abline(-log10(0.10),1, col='black',lty=1)
    text(0.5, 1.6, "FDR=0.10", col = "gray60",srt=35, cex=1)
    abline(-log10(0.25),1, col='black',lty=1)
    text(0.5, 1.2, "FDR=0.25", col = "gray60",srt=35, cex=1)
    #legend('topleft', c("FDR = 0.05","FDR = 0.10","FDR = 0.25"),
           #col=c('black','black','black'),lty=c(1,1,1), cex=0.8)
    if (BF)
    {
      abline(h=-log10(0.05/nn), col='black') ## bonferroni
    }
  }
}

#plot_7249 contains a set of glucose response SNPs META P values for previously done META analysis of GOKIND and EDIC cohorts, the complete set of SNPs from that META analysis can be found in META_pvalues_combined_final

fin7253=read.table("plot_7249", header=T)
qqunif(fin7253$META_pval)
```
