# Supplementary material for: Integration of genomics and transcriptomics predicts diabetic retinopathy susceptibility genes
Source: eLife. 2020 Nov 9;9:e59980. doi: 10.7554/eLife.59980 (PMC7728435; doi:10.7554/eLife.59980)
Supplement: Source code 8. [file elife-59980-code8.zip › Sourcecode8.pdf]

```
library("ggplot2")
library(lattice)
library(ggsignif)


## THIS DATA WAS CREATED IN THE FILE GWAS_EQTL_ENRICHEMTN.R ##
GWAS <- read.table(file = "GWAS_eQTL_ind.txt", as.is=T, header=T)
#get rid of mendelian columns
g=GWAS[, -grep(".mend", colnames(GWAS))]
GWAS1=g

## PLOT ENRICHEMENT FOR INTERESTING GENES ONLY ##
interesting.gene.eqtls <- read.table(file = "rg_pdr_npdr_gene_vs_GTex_v7_Full.csv", as.is=T,
                                     header=T, sep=",")
interesting.gene.eqtls$snp <- gsub("_[A-Z].*","", interesting.gene.eqtls$variant_id)
interesting.gene.eqtls$gene_id <- gsub("\\..*","",interesting.gene.eqtls$gene_id)
interesting.genes <- unique(interesting.gene.eqtls$gene_id)

ind <- which(GWAS1$chrpos %in% interesting.gene.eqtls$snp |
             GWAS1$rs %in% interesting.gene.eqtls$rs_id_dbSNP147_GRCh37p13)
GWAS1$int.gene.eqtl <- 0
GWAS1$int.gene.eqtl[ind] <- 1

m=merge(GWAS1,interesting.gene.eqtls, by.x = "chrpos", by.y = "snp")

## DETERMINE THE PROPORTION OF P-VALUES < .05 IN
#1) FULL GWAS 2) ALL EQL IN QWAS 3) EQTL OF 103 GENES IN GWAS
## 1)
gwas.all.p.05 = mean(GWAS1$META_pval <= .05, na.rm=T)
## 3)
ind <- GWAS1$chrpos %in% interesting.gene.eqtls$snp
gwas.103.p.05 <- mean(GWAS1$META_pval[ind] < 0.05)

## 2) change 54 to 101
eqtl.ind <- rowSums(GWAS1[,6:54]==1, na.rm=T)>0
gwas.eqtl.p.05 <- mean(GWAS1$META_pval[eqtl.ind] < 0.05, na.rm=T)

toplot <- data.frame( cat=c("All SNPs", "All eQTL", "eQTL from 103 genes"),
                      props =c(gwas.all.p.05, gwas.eqtl.p.05, gwas.103.p.05))
ses.gwas.all <- sqrt(gwas.all.p.05 * (1 - gwas.all.p.05) / sum(!is.na(GWAS$META_pval)))
ses.gwas.103.p.05 <- sqrt(gwas.103.p.05 * (1 - gwas.103.p.05 ) / sum(ind))
ses.gwas.eqtl.p.05 <- sqrt(gwas.eqtl.p.05 * (1- gwas.eqtl.p.05) / sum(eqtl.ind))
```

```
toplot$ses <- c(ses.gwas.all, ses.gwas.eqtl.p.05, ses.gwas.103.p.05)

mean.comp <- data.frame(.y. = c("len","len","len"), group1 = c("All SNPs", "All SNPs", "All
eQTL"),
                        group2 = c("All eQTL", "eQTL from 103 genes", "eQTL from 103 genes"),
                        p = c(.01, .05, .001),
                        p.adj = c(.01, .05, .001),
                        p.format = c(.01, .05, .001),
                        p.signif = c("*","*","*"),
                        method = c("Wald","Wald","Wald"),
                        y.pos = c(0.06, 0.065, 0.070))


# calculating P values for the plot

diffs <-  c(toplot$props[1] - toplot$props[2],
            toplot$props[2] - toplot$props[3],
            toplot$props[1] - toplot$props[3])
vars <- c(toplot$ses[1]^2 + toplot$ses[2]^2,
          toplot$ses[2]^2 + toplot$ses[3]^2,
          toplot$ses[1]^2 + toplot$ses[3]^2)
z <- diffs/sqrt(vars)
ps <- (1 - pnorm(abs(z)))*2

options(digits=3)

ax.11.text <- element_text(size = 10)
ay.11.text <- element_text(size = 10)
p<-ggplot(data=toplot, aes(x=cat, y=props)) +
  geom_bar(stat="identity",width=0.5, fill="steelblue")+
  geom_errorbar(aes(ymin=props-1.96*ses, ymax=props+1.96*ses), width=.1,
                position=position_dodge(.9))  +

  geom_signif(comparisons=list( c("All eQTL", "eQTL from 103 genes"),
                              c("All SNPs", "eQTL from 103 genes")),
              y_position=c(0.065, 0.07), tip_length=0, annotation=c("p = 0.0012", "p = 0.0023")) +
  scale_y_continuous(breaks=seq(0,.06,by=.01)) +
  xlab("") + ylab("Proportion p-values < 0.05") +
  theme_classic()+
  theme(panel.grid.major.x = element_line(size = 0.1, color = "grey"),
        panel.grid.major.y = element_blank(),
        panel.grid.minor = element_blank(),axis.text.x = ax.11.text,axis.text.y=ay.11.text
```

)

p
