# Supplementary material for: Integration of genomics and transcriptomics predicts diabetic retinopathy susceptibility genes
Source: eLife. 2020 Nov 9;9:e59980. doi: 10.7554/eLife.59980 (PMC7728435; doi:10.7554/eLife.59980)
Supplement: Source code 9. [file elife-59980-code9.zip › Sourcecode9.pdf]

```
#get all Retina genes for all chromosomes and extract those with FDR<=0.05

#for retina
#!/bin/bash
for f in Retina.nominal.eQTL.chr*.with_thresholds.txt
do
   awk '(NR>1) && ($15 <= 0.05 )' "$f" > "temp_file" && mv "temp_file" "$f"
done

#join files into one: Retina.nominal.eQTL.ALL.with_thresholds.txt

a=fread("Retina.nominal.eQTL.ALL.with_thresholds.txt")
> c=select(a,V1)
> c=na.omit(c)
c=unique(c)
write.table(c, file="Retina.v7.egenes.txt",  sep = " ", row.names = FALSE, col.names =
FALSE,quote=FALSE)

# parse all analyzed 12503 genes among GTEX tissues (and apply FDR<=0.05)

#!/bin/bash
for f in *.v7.egenes.txt
do
   awk '(NR>1) && ($29 <= 0.05 )' "$f" > "temp_file" && mv "temp_file" "$f"
done


cd /projects/com_grassim/anamaria/anamaria/anamaria/GTExV7
awk 'NR==FNR { gene[$1]=1 ; next } gene[$1]' allGenes.txt *.v7.egenes.txt > output2.txt
awk 'NR==FNR { gene[$1]=1 ; next } gene[$1]' sig_130genes.txt  *.v7.egenes.txt >
output103_2.txt

10831  for all analyzed genes, 10831/12503=0.87
103 for 103

#make plot

a=fread("output2.txt",fill=TRUE)
b=a %>% group_by(V1) %>% summarise(col3=n_distinct(V12))
b=as.data.frame(b)
write.table(b, file="allG.txt",  sep = " ", row.names = FALSE, col.names = FALSE,quote=FALSE)

a=fread("output103_2.txt",fill=TRUE)
```

```
b=a %>% group_by(V1) %>% summarise(col3=n_distinct(V12))
b=as.data.frame(b)
write.table(b, file="number_of_eqtles_per_gene_103genes_all26850.txt",  sep = " ", row.names
= FALSE, col.names = FALSE,quote=FALSE)

b=read.table("number_of_eqtles_per_gene_103genes_all26850.txt", header=T)
a=read.table("allG.txt", header=F)

wilcox.test(a$V2, y = b$number_of_eqtles_per_gene,paired = FALSE)
t.test(a$V2, y = b$number_of_eqtles_per_gene)

df <- data.frame("prop" = c(0.87,1), "Name" = c("All Genes \n 12503","Glucose Response
Genes \n 103"))

p <- ggplot(data = df, aes(x = Name, y = prop, fill = Name)) +
  geom_bar(stat = "identity") +
  labs(x = "", y = "Proportion of eGenes") +
  scale_fill_brewer(palette="Greens",name = "Number of cis EQTL", labels = c("3124345",
"26846")) +
  theme_minimal() +
  theme(legend.position = "right",
        panel.grid.major.y = element_blank(),
        panel.grid.minor.y = element_blank(),
        axis.line = element_line(),
        axis.ticks = element_line())

p + ggplot2::annotate("text", x = 1.5, y = 1.2, label = "p < 2e-16", size = 3.5) +
  ggplot2::annotate("rect", xmin = 1, xmax = 2, ymin = 1.1, ymax =1.1, alpha=0.3,colour =
"black")
```
