# Supplementary material for: Integration of genomics and transcriptomics predicts diabetic retinopathy susceptibility genes
Source: eLife. 2020 Nov 9;9:e59980. doi: 10.7554/eLife.59980 (PMC7728435; doi:10.7554/eLife.59980)
Supplement: Source code 10. [file elife-59980-code10.zip › Sourcecode10.pdf]

```
setwd("/projects/com_grassim/anamaria/anamaria/anamaria/plot")
library("ggplot2")
library(lattice)
library(qqman)
source("manhattan_plot.r")
source("qq_plot.r")

PLOTDIR <- "/projects/com_grassim/anamaria/anamaria/anamaria/plot/"

## THIS DATA WAS CREATED IN THE FILE GWAS_EQTL_ENRICHEMTN.R ##
GWAS <- read.table(file = "GWAS_eQTL_ind.txt", as.is=T, header=T)

## PLOT ENRICHEMENT FOR INTERESTING GENES ONLY ##
interesting.gene.eqtls <- read.table(file = "rg_pdr_npdr_gene_vs_GTex_v7_Full.csv", as.is=T,
                                     header=T, sep=",")
interesting.gene.eqtls$snp <- gsub("_[A-Z].*","", interesting.gene.eqtls$variant_id)
interesting.gene.eqtls$gene_id <- gsub("\\..*","",interesting.gene.eqtls$gene_id)
interesting.genes <- unique(interesting.gene.eqtls$gene_id)

ind <- which(GWAS$chrpos %in% interesting.gene.eqtls$snp |
             GWAS$rs %in% interesting.gene.eqtls$rs_id_dbSNP147_GRCh37p13)
GWAS$int.gene.eqtl <- 0
GWAS$int.gene.eqtl[ind] <- 1

## DETERMINE WHICH SNPS ARE EQTLS FOR WHICH TISSUES ##
#GTEX.DIR <- "/gpfs/data/stranger-lab/askol/GTEx/GTEx_Analysis_v7_eQTL/"
GTEX.DIR <-"/projects/com_grassim/anamaria/anamaria/anamaria/plot/gtext/"
files <- dir(GTEX.DIR, pattern="v7.signif")


gwas.newnames <- read.table(file ="GWAS_newnames.txt", as.is=T, header=T)

GWAS <- gwas.newnames

gene.snps <- c()
for (file in files){

   tissue <- gsub("\\..*","",file)
   print(tissue)

   ## READ IN SNPS NAMES ##
   cmd <- paste0("awk '{print $1,$2,$7}' ", GTEX.DIR,file)
   #cmd <- paste0("zcat ",GTEX.DIR,file, "| 'awk '{print $1,$2,$7}' ")
```

```
    snps <- system(cmd, intern=T)
    snps <- do.call(rbind, strsplit(snps, split=" "))
    snps[,1] <- sub("_[A-Z].*","", snps[,1])
    snps[,2] <- sub("\\..*","",snps[,2])
    snps <- snps[-1,]

    if (file == files[1]){
        gene.snps <- paste(snps[,1],snps[,2],sep=".")
    }else{
        gene.snps.tmp <-   paste(snps[,1],snps[,2],sep=".")
        gene.snps <- c(gene.snps, gene.snps.tmp[gene.snps.tmp %in% gene.snps == FALSE])
    }

}


genes <- gsub(".*\\.","",gene.snps)
snps <- gsub("\\..*", "", gene.snps)


GS <- data.frame(gene = genes, snp = snps)

## REMOVE SNPS NOT IN GWAS
rm <- which(GS$snp %in% GWAS$chrpos == FALSE)
GS <- GS[-rm,]

uniq.genes <- unique(GS$gene)
## GS <- merge(GS, GWAS[,c("chrpos","META_pval")], by.x = "snp", by.y="chrpos", keep.x=T)
GS <- merge(GS, GWAS[,c("chrpos","META_pval","PVALUE.1")], by.x = "snp", by.y="chrpos",
keep.x=T)

snp.idx <- list()
for (gene in uniq.genes){
    snp.idx[[gene]] <- which(GS$gene == gene)
}

save.image(file = "HakaeFig6.RData")
## save.image(file = "HakaeFig6_EDIC.RData")



## CHOOSE 103 GENES AND ASK WHAT NUMBER AND PROPORTION OF SNPS HAVE
q-VALUES < .05 ##
```

```
perm.sign.snps.meta <- perm.sign.snps.edic <- c()
n.genes.sample = 103
for (i in 1:10000){

   if (i%%500==0){print(i)}
   genes.perm <- as.character(sample(uniq.genes, n.genes.sample))
   snps <- c()
   pos <- unique(unlist(lapply(genes.perm, function(x) snp.idx[[x]])))
   #ps.ind.meta <- (GS$META_pval[pos]<= .05)*1
   #ps.ind.edic <- (GS$PVALUE.1[pos] <= .05)*1
   ps.ind.meta <- (p.adjust(GS$META_pval[pos], method="BH") <= .05)*1
   ps.ind.edic <- (p.adjust(GS$PVALUE.1[pos], method="BH") <= .05)*1
   perm.sign.snps.meta <- rbind(perm.sign.snps.meta,
                                c(sum(!is.na(ps.ind.meta)),
                                 sum(ps.ind.meta, na.rm=T), mean(ps.ind.meta, na.rm=T)))
   perm.sign.snps.edic <- rbind(perm.sign.snps.edic,
                                c(sum(!is.na(ps.ind.edic)),
                                 sum(ps.ind.edic, na.rm=T), mean(ps.ind.edic, na.rm=T)))
}

perm.sign.snps.meta <- data.frame(n=perm.sign.snps.meta[,1], count=perm.sign.snps.meta[,2],
                                  prop=perm.sign.snps.meta[,3])
perm.sign.snps.edic <- data.frame(n=perm.sign.snps.edic[,1], count=perm.sign.snps.edic[,2],
                                  prop=perm.sign.snps.edic[,3])

write.table(file = "perm.sign.snps.meta", perm.sign.snps.meta, quote=F, row.names=F,
col.names=T)
write.table(file = "perm.sign.snps.edic", perm.sign.snps.edic, quote=F, row.names=F,
col.names=T)


ind <- which(GWAS$int.gene.eqtl == 1)
for (i in 1:2){

   name.ext=""
   if (i == 1){
      GWAS.col <- grep("META_pval", names(GWAS))
      perm.sign.snps <- perm.sign.snps.meta
      name.ext = "Meta"
   }else{
      GWAS.col <- grep("PVALUE.1", names(GWAS))
      perm.sign.snps <- perm.sign.snps.edic
```

```
        name.ext="EDIC"
    }

    ps.ind <- (p.adjust(GWAS[ind, GWAS.col], method="BH") <= 0.05)*1
    obs <- c(sum(!is.na(ps.ind)), sum(ps.ind, na.rm=T), mean(ps.ind, na.rm=T))
    obs <- data.frame(x=obs[3], y=0.03)


    pdf(paste0(PLOTDIR,"Hist_Exp_GWAS_lt05_",name.ext,".pdf"))
    my.text <- element_text(size=14)
    p<-ggplot(perm.sign.snps, aes(x=prop)) +
        geom_histogram(color="black", fill="white") +
            xlab("Proportion of SNPs (q-value < 0.05)") +
                ylab("Number of Simulations") +
                    coord_cartesian(ylim = c(0, 500)) +
                        geom_point(data=obs, aes(x=x, y=y), color="red", size=6) +
                            theme_bw() + theme(axis.title = my.text, axis.text=my.text)
    print(p)

    ## WITHOUT THE 500 COUNT LIMIT ON Y AXIS
    p<-ggplot(perm.sign.snps, aes(x=prop)) +
        geom_histogram(color="black", fill="white") +
            xlab("Proportion of SNPs (q-value < 0.05)") +
                ylab("Number of Simulations") +
                        geom_point(data=obs, aes(x=x, y=y), color="red", size=6) +
                            theme_bw() + theme(axis.title = my.text, axis.text=my.text)
    print(p)


    1 - sum(obs$x >= perm.sign.snps$prop)/nrow(perm.sign.snps)
    dev.off()
}

save.image(file = "HakaeFig6.RData")
```
