# Supplementary material for: Integration of genomics and transcriptomics predicts diabetic retinopathy susceptibility genes
Source: eLife. 2020 Nov 9;9:e59980. doi: 10.7554/eLife.59980 (PMC7728435; doi:10.7554/eLife.59980)
Supplement: Source code 11. [file elife-59980-code11.zip › Sourcecode11.pdf]

```
#get files from here:
ftp://ftp.ncbi.nlm.nih.gov/snp/organisms/human_9606_b150_GRCh37p13/VCF/

module load tools/gtc2vcf

#META_pvalues_combined_final comes from previous META analysis of Gokind and EDIC
cohorts

awk '{ print $1}' META_pvalues_combined_final > META_rs
awk '{ print $1,$4}' META_pvalues_combined_final > META_pvalues

vcftools --gzvcf 00-All.vcf.gz --snps META_rs --recode --out  META_RSID


awk '{ print $1,$2, $3 }' META_RSID.recode.vcf > META_RSID
sed '/^#/d' META_RSID > META_RSID.F

library(qqman)
library(data.table)

a=fread("META_pvalues", header=T)
b=fread("META_RSID.F", header=F)
colnames(b)=c("CHR","POS","MARKER")
m=merge(a,b,by="MARKER")
m1=na.omit(m)
write.table(m1, file="meta_b37_chr_pos_rsid",  sep = " ", row.names = FALSE, col.names =
TRUE,quote=FALSE)

#manhattn_7253 is 7253 Glucose response EQTLS from META analysis taken from
meta_b37_chr_pos_rsid

df=fread("manhattn_7253", header=T)
manhattan(df, chr="CHR", bp="POS", snp="SNP", p="METAP",annotatePval = 1e-5,ylim=c(0,6))
```
