# Supplementary material for: Integration of genomics and transcriptomics predicts diabetic retinopathy susceptibility genes
Source: eLife. 2020 Nov 9;9:e59980. doi: 10.7554/eLife.59980 (PMC7728435; doi:10.7554/eLife.59980)
Supplement: Source code 12. [file elife-59980-code12.zip › Sourcecode12.pdf]

```
library("ggplot2")
library(lattice)
library(ggsignif)

## THIS DATA WAS CREATED IN THE FILE GWAS_EQTL_ENRICHEMTN.R ##
GWAS <- read.table(file = "GWAS_eQTL_ind.txt", as.is=T, header=T)
#get rid of mendelian columns
g=GWAS[, -grep(".mend", colnames(GWAS))]
GWAS1=g

## PLOT ENRICHEMENT FOR INTERESTING GENES ONLY ##
interesting.gene.eqtls <- read.table(file = "rg_pdr_npdr_gene_vs_GTex_v7_Full.csv", as.is=T,
                              header=T, sep=",")
interesting.gene.eqtls$snp <- gsub("_[A-Z].*","", interesting.gene.eqtls$variant_id)
interesting.gene.eqtls$gene_id <- gsub("\\..*","",interesting.gene.eqtls$gene_id)
interesting.genes <- unique(interesting.gene.eqtls$gene_id)

ind <- which(GWAS1$chrpos %in% interesting.gene.eqtls$snp |
           GWAS1$rs %in% interesting.gene.eqtls$rs_id_dbSNP147_GRCh37p13)

# 0)
eqtl_fn =read.table("locus_retina_gwas_zoom", header=T)
gtx=eqtl_fn$pval

## 1)
# 272 eQTLs for >20 tissues and Retina for FLCN
c=read.table("FLCN_allTissuesAndRetina_together_466_METAG", header=T)
cc=c[!duplicated(c), ]
flcn=cc$META_pval

# get rid of mendelian in GWAS
g=GWAS[, -grep(".mend", colnames(GWAS))]

## 2) all SNP
gwas.all.p.N = g$META_pval
gwas.all.p.N1 <- gwas.all.p.N[!is.na(gwas.all.p.N)]

## 3) eQTLs from 103 genes, the issue is here
ind <- g$chrpos %in% interesting.gene.eqtls$snp
gwas.103.p.N <- g$META_pval[ind]

## 4) all eQTLS
eqtl.ind <- rowSums(g[,6:54]==1, na.rm=T)>0
```

```
gwas.eqtl.p.N <- g$META_pval[eqtl.ind]
gwas.eqtl.p.N1=gwas.eqtl.p.N[!is.na(gwas.eqtl.p.N)]

ptresh=0.05

FDR0=qvalue(gtx,pi0 = 1)
FDR1=qvalue(flcn)
FDR2=qvalue(gwas.all.p.N1,pi0=1) # added pi0=1 for this plot
FDR3=qvalue(gwas.103.p.N)
FDR4=qvalue(gwas.eqtl.p.N1)


FDR0FI=1-((FDR0$pi0)*ng0*ptresh)/sum(gtx<ptresh)
FDR1FI=1-((FDR1$pi0)*ng1*ptresh)/sum(flcn<ptresh)
FDR2FI=1-((FDR2$pi0)*ng2*ptresh)/sum(gwas.all.p.N1<ptresh)
FDR3FI=1-((FDR3$pi0)*ng3*ptresh)/sum(gwas.103.p.N<ptresh)
FDR4FI=1-((FDR4$pi0)*ng4*ptresh)/sum(gwas.eqtl.p.N1<ptresh)


toplot.N <- data.frame( set=c("FLCN", "All SNPs", "eQTL from 103 genes","All eQTL"),
                        FDR =c(FDR1FI,FDR2FI,FDR3FI,FDR4FI))

toplot.N$set <- factor(toplot.N$set,
                       levels = c("All SNPs", "All eQTL", "eQTL from 103 genes", "FLCN"))

p<-ggplot(toplot.N, aes(x=set, y=FDR, fill=set)) + labs(y = "TPR")+
  geom_bar(stat="identity")+theme(axis.title.x = element_blank())+
  geom_hline(yintercept=0.05, linetype="dashed", color = "red")
p
```
