# Supplementary material for: Integration of genomics and transcriptomics predicts diabetic retinopathy susceptibility genes
Source: eLife. 2020 Nov 9;9:e59980. doi: 10.7554/eLife.59980 (PMC7728435; doi:10.7554/eLife.59980)
Supplement: Source code 13. [file elife-59980-code13.zip › Sourcecode13.pdf]

```
{\rtf1\ansi\ansicpg1252\cocoartf1404\cocoasubrtf470
{\fonttbl\f0\fswiss\fcharset0 Helvetica;}
{\colortbl;\red255\green255\blue255;}
\margl1440\margr1440\vieww10800\viewh8400\viewkind0
\pard\tx720\tx1440\tx2160\tx2880\tx3600\tx4320\tx5040\tx5760\tx6480\tx7200\tx7920\tx8640\pardirnatural\partightenfactor0

\f0\fs24 \cf0 library(data.table)\
library(ggplot2)\
\
## read RG_PDR_nPDR\
HOME <- "/Users/ams/Desktop/Grassi/qpcr/"  # change if needed\
source(paste(HOME,"func.R",sep=""))\
\
dat<-local(get(load(file=paste(HOME,"normalizedDataMatrix_filtered.RData",sep=""))))\
\
probeList <- rownames(dat)  # probeID (nuID) gene annotation\
\
if (require(lumiHumanAll.db) & require(annotate))\{\
  geneSymbol<- getSYMBOL(probeList, 'lumiHumanAll.db')\
  geneName<- sapply(lookUp(probeList, 'lumiHumanAll.db', 'GENENAME'), function(x) x[1])\
\}\
genes <-data.frame(ID=probeList, geneSymbol=geneSymbol, geneName=geneName, stringsAsFactors=FALSE)\
target_gene="FLCN"\
\
#  nPDR\
subject<-"No_PDR"\
DwoC<-dat[,grep("DwoC",colnames(dat))] # grep all normal samples\
```

```
DwoC_sg<-DwoC[,grep("norm",colnames(DwoC))]; DwoC_hg<-DwoC[,grep("30mM",colnames(DwoC))];\
cname=c("nPDR1","nPDR2","nPDR3","nPDR4","nPDR5","nPDR6","nPDR7")\
DwoC_delta <- DwoC_hg/DwoC_sg; \
\
DwoC_sg_avg<-prep_data(DwoC_sg); \
colnames(DwoC_sg_avg)<-cname\
DwoC_hg_avg<-prep_data(DwoC_hg); \
colnames(DwoC_hg_avg)<-cname\
DwoC_delta_avg<-prep_data(DwoC_delta); \
colnames(DwoC_delta_avg)<-cname\
#rm(DwoC, DwoC_sg, DwoC_hg, DwoC_delta)\
\
\
DwoC_delta_avg_FLCN<-DwoC_delta_avg[which(genes$geneSymbol==target_gene),]\
\
\
\
# 3. PDR\
cname2=c("PDR1","PDR2","PDR3","PDR4","PDR5","PDR6","PDR7","PDR8")\
DwC<-dat[,grep("DwC",colnames(dat))] # grep all normal samples\
DwC_sg<-DwC[,grep("norm",colnames(DwC))]; DwC_hg<-DwC[,grep("30mM",colnames(DwC))];\
DwC_delta <- DwC_hg/DwC_sg\
DwC_sg_avg<-prep_data(DwC_sg)\
colnames(DwC_sg_avg)<-cname2\
DwC_hg_avg<-prep_data(DwC_hg)\
colnames(DwC_hg_avg)<-cname2\
DwC_delta_avg<-prep_data(DwC_delta)\
colnames(DwC_delta_avg)<-cname2\
#rm(DwC, DwC_sg, DwC_hg, DwC_delta)\
```

```
\

\

DwC_delta_avg_FLCN<-DwC_delta_avg[which(genes$geneSymbol==target_gene),]\

\

delta<-as.data.frame(t(cbind(DwC_delta_avg_FLCN,DwoC_delta_avg_FLCN)))\

delta$subject<-
c("PDR","PDR","PDR","PDR","PDR","PDR","PDR","PDR","nDR","nDR","nDR","nDR","nDR","nDR","nDR")\

delta$subject1<-rownames(delta)\

colnames(delta)<-c("FLCN1","FLCN2","FLCN3","subject", "subject1")\

\

boxplot(FLCN3~subject,data=delta,xlim=c(0.8,5.2),ylim=c(0.9,1.1),col =
c("palevioletred1","royalblue1"),xlab="subjects",ylab="Expression estimate in delta (log2)",boxwex =
0.2,frame.plot = FALSE)\

stripchart(FLCN3~subject, vertical = TRUE, data = delta,method = "jitter", add = TRUE,pch = 20,
col=rgb(0,0,0,.5),jitter = 0.001)}
```
