# Supplementary material for: Integration of genomics and transcriptomics predicts diabetic retinopathy susceptibility genes
Source: eLife. 2020 Nov 9;9:e59980. doi: 10.7554/eLife.59980 (PMC7728435; doi:10.7554/eLife.59980)
Supplement: Supplementary file 1. — Demographic features of the DCCT/EDIC study subjects with type 1 diabetes. Source files can be found at Epidemiology of Diabetes Interventions and Complications (EDIC). Design, implementation, and preliminary results of a long-term follow-up of the Diabetes Control and Complications Trial cohort. Diabetes Care. 1999 Jan;22(1):99–111. pmid:10333910. Pubmed Central PMCID: 2745938. Epub 1999/05/20. eng. Supplementary file 1b. Differential response to Glucose PDR vs. nDR (RGpdr–ndr). Source file for this table is available at Source data 1. Supplementary file 1c. Demographic features of UK Biobank subjects with diabetes used in the diabetic retinopathy GWAS. Source file for this table is available at https://www.ukbiobank.ac.uk/. Supplementary file 1d. Demographic features of individuals without diabetes from the Coriell Institute for Medical Research NIGMS Human Genetic Cell Repository. Source file for this table is available at Coriell Institute for Medical Research NIGMS Human Genetic Cell Repository (http://ccr.coriell.org/). [file elife-59980-supp1.docx]

**Supplementary File 1**

**Supplementary file 1a. Demographic features of the DCCT/EDIC study subjects with type 1 diabetes**

|  | **nDR** | **PDR** |
| --- | --- | --- |
| **N** | **7** | **8** |
| **Mean age in years at DCCT baseline* (yrs, std)** | **32 (3.9)** | **31 (8.5)** |
| **Caucasian Ethnicity (%)** | **100** | **100** |
| **Female (%)** | **4 (57.1)** | **5 (62.5)** |
| **Mean duration of type 1 diabetes in months at DCCT baseline (yrs, std)** | **27 (13.4)** | **53 (43.4)** |
| **HbA1c^+^ (%, std)** | **7.62 (1.07)** | **9.71 (2.37)** |
| **Intensive Treatment group^ (%)** | **2 (28.6)** | **3 (37.5)** |
| **Secondary Intervention Cohort# (%)** | **0 (0)** | **1 (12.5)** |

All subjects from DCCT/EDIC – the Diabetes Control and Complications Trial/ Epidemiology of Diabetes Interventions and Complications cohort

nDR - no Diabetic Retinopathy; PDR - proliferative diabetic retinopathy

* DCCT baseline at subject enrollment (1983–1989)

(std) standard deviation

^+^HbA1c mean includes all DCCT visits except the baseline visit.

^ For the duration of the DCCT study the intensive treatment group maintained a HbA1c of

approximately 7% as compared to approximately 9% in the conventional treatment group.

#The Secondary Intervention Cohort consisted of subjects with type 1 diabetes for 1-15 years

and mild to moderate non-proliferative retinopathy and a urinary albumin excretion rate < 200

mg/dl at baseline. The Primary Prevention cohort consisted of subjects with type 1 diabetes for 1-5 years

and no diabetes related complications.

**Supplementary file 1b. Differential response to Glucose PDR vs nDR (RG_pdr-ndr_)**

| **Gene Symbol** | **Gene Name** | **log2FC** | **P.Value** |
| --- | --- | --- | --- |
| **RASA3** | **RAS p21 protein activator 3** | **-0.26** | **3.1x10^-5^** |
| **HBQ1** | **hemoglobin subunit theta 1** | **-0.39** | **7.6x10^-5^** |
| **RIMKLA** | **ribosomal modification protein rimK like family member A** | **0.41** | **2x10^-4^** |
| **FMC1** | **formation of mitochondrial complex V assembly factor 1 homolog** | **-0.16** | **4x10^-4^** |
| **MMADHC** | **methylmalonic aciduria and homocystinuria, cblD type** | **0.13** | **4x10^-4^** |
| **ARFGEF2** | **ADP ribosylation factor guanine nucleotide exchange factor 2** | **0.15** | **4.8x10^-4^** |
| **ADAM23** | **ADAM metallopeptidase domain 23** | **-0.31** | **7.9x10^-4^** |
| **TRAPPC8** | **trafficking protein particle complex 8** | **0.13** | **7.9x10^-4^** |
| **SPIRE1** | **spire type actin nucleation factor 1** | **0.13** | **1x10^-3^** |
| **KCNK3** | **potassium two pore domain channel subfamily K member 3** | **0.25** | **1.1x10^-3^** |
| **RNF126P1** | **ring finger protein 126 pseudogene 1** | **0.23** | **1.2x10^-3^** |
| **CWC22** | **CWC22 spliceosome associated protein homolog** | **0.17** | **1.2x10^-3^** |
| **CPA4** | **carboxypeptidase A4** | **-0.26** | **1.2x10^-3^** |
| **CEL** | **carboxyl ester lipase** | **-0.22** | **1.4x10^-3^** |
| **CENPH** | **centromere protein H** | **-0.27** | **1.4x10^-3^** |
| **UCA1** | **urothelial cancer associated 1 (non-protein coding)** | **0.26** | **1.4x10^-3^** |
| **METTL17** | **methyltransferase like 17** | **-0.13** | **1.5x10^-3^** |
| **CYTIP** | **cytohesin 1 interacting protein** | **0.12** | **1.5x10^-3^** |
| **CSNK2A1** | **casein kinase 2 alpha 1** | **0.16** | **1.6x10^-3^** |
| **SOWAHC** | **sosondowah ankyrin repeat domain family member C** | **0.23** | **1.9x10^-3^** |
| **SLC48A1** | **solute carrier family 48 member 1** | **0.27** | **2x10^-3^** |
| **TXLNG** | **taxilin gamma** | **0.22** | **2.1x10^-3^** |
| **FAM219B** | **family with sequence similarity 219 member B** | **0.13** | **2.1x10^-3^** |
| **FAM107B** | **family with sequence similarity 107 member B** | **0.16** | **2.1x10^-3^** |
| **NAA30** | **N(alpha)-acetyltransferase 30, NatC catalytic subunit** | **0.15** | **2.4x10^-3^** |
| **COG4** | **component of oligomeric golgi complex 4** | **-0.14** | **2.4x10^-3^** |
| **RC3H2** | **ring finger and CCCH-type domains 2** | **-0.12** | **2.5x10^-3^** |
| **CARMIL2** | **capping protein regulator and myosin 1 linker 2** | **-0.17** | **2.5x10^-3^** |
| **FLCN** | **folliculin** | **0.27** | **2.5x10^-3^** |
| **RNF115** | **ring finger protein 115** | **0.12** | **2.7x10^-3^** |
| **RGPD1** | **RANBP2-like and GRIP domain containing 1** | **0.42** | **2.8x10^-3^** |
| **ABHD11** | **abhydrolase domain containing 11** | **-0.17** | **2.9x10^-3^** |
| **TMEM161A** | **transmembrane protein 161A** | **0.23** | **3x10^-3^** |
| **HIST1H2AB** | **histone cluster 1 H2A family member b** | **-0.19** | **3x10^-3^** |
| **AKTIP** | **AKT interacting protein** | **0.10** | **3.1x10^-3^** |
| **PKNOX1** | **PBX/knotted 1 homeobox 1** | **-0.11** | **3.1x10^-3^** |
| **NT5C3B** | **5'-nucleotidase, cytosolic IIIB** | **-0.09** | **3.3x10^-3^** |
| **RNF7** | **ring finger protein 7** | **0.09** | **3.3x10^-3^** |
| **HSPA1A** | **heat shock protein family A (Hsp70) member 1A** | **0.21** | **3.4x10^-3^** |
| **MT1A** | **metallothionein 1A** | **-0.21** | **3.5x10^-3^** |
| **TNFRSF18** | **TNF receptor superfamily member 18** | **0.25** | **3.6x10^-3^** |
| **STAP2** | **signal transducing adaptor family member 2** | **0.21** | **3.9x10^-3^** |
| **IL12RB2** | **interleukin 12 receptor subunit beta 2** | **0.25** | **4x10^-3^** |
| **NUDT16P1** | **nudix hydrolase 16 pseudogene 1** | **-0.19** | **4.1x10^-3^** |
| **LSM7** | **LSM7 homolog, U6 small nuclear RNA and mRNA degradation associated** | **-0.09** | **4.1x10^-3^** |
| **PHF6** | **PHD finger protein 6** | **0.19** | **4.3x10^-3^** |
| **ARHGEF19** | **Rho guanine nucleotide exchange factor 19** | **-0.17** | **4.4x10^-3^** |
| **EXO1** | **exonuclease 1** | **-0.14** | **4.6x10^-3^** |
| **UBE2G1** | **ubiquitin conjugating enzyme E2 G1** | **-0.10** | **4.7x10^-3^** |
| **HAUS4** | **HAUS augmin like complex subunit 4** | **-0.11** | **4.8x10^-3^** |
| **KANSL1L** | **KAT8 regulatory NSL complex subunit 1 like** | **-0.21** | **4.9x10^-3^** |
| **RABGGTB** | **Rab geranylgeranyltransferase beta subunit** | **0.12** | **4.9x10^-3^** |
| **XRCC1** | **X-ray repair cross complementing 1** | **-0.12** | **4.9x10^-3^** |
| **ZHX1** | **zinc fingers and homeoboxes 1** | **0.12** | **5x10^-3^** |
| **CAPN2** | **calpain 2** | **-0.22** | **5.2x10^-3^** |
| **ARID4A** | **AT-rich interaction domain 4A** | **0.19** | **5.2x10^-3^** |
| **ZNF222** | **zinc finger protein 222** | **0.21** | **5.2x10^-3^** |
| **PKN1** | **protein kinase N1** | **-0.14** | **5.6x10^-3^** |
| **YWHAG** | **tyrosine 3-monooxygenase/tryptophan 5-monooxygenase activation protein gamma** | **0.15** | **5.6x10^-3^** |
| **PRKCA** | **protein kinase C alpha** | **0.24** | **5.6x10^-3^** |
| **IKZF1** | **IKAROS family zinc finger 1** | **0.18** | **5.7x10^-3^** |
| **JMY** | **junction mediating and regulatory protein, p53 cofactor** | **0.18** | **5.7x10^-3^** |
| **MPLKIP** | **M-phase specific PLK1 interacting protein** | **0.18** | **5.7x10^-3^** |
| **ISG15** | **ISG15 ubiquitin-like modifier** | **-0.11** | **5.8x10^-3^** |
| **GSDME** | **gasdermin E** | **-0.10** | **5.9x10^-3^** |
| **IL17D** | **interleukin 17D** | **-0.21** | **5.9x10^-3^** |
| **AFG3L2** | **AFG3 like matrix AAA peptidase subunit 2** | **-0.11** | **6x10^-3^** |
| **LSM14B** | **LSM family member 14B** | **0.22** | **6.2x10^-3^** |
| **PPP1CA** | **protein phosphatase 1 catalytic subunit alpha** | **-0.13** | **6.3x10^-3^** |
| **FILIP1L** | **filamin A interacting protein 1 like** | **0.19** | **6.3x10^-3^** |
| **XRRA1** | **X-ray radiation resistance associated 1** | **0.23** | **6.3x10^-3^** |
| **MKI67** | **marker of proliferation Ki-67** | **-0.15** | **6.4x10^-3^** |
| **AGPAT2** | **1-acylglycerol-3-phosphate O-acyltransferase 2** | **-0.16** | **6.4x10^-3^** |
| **IFI27** | **interferon alpha inducible protein 27** | **-0.20** | **6.5x10^-3^** |
| **PADI4** | **peptidyl arginine deiminase 4** | **-0.29** | **6.5x10^-3^** |
| **HYOU1** | **hypoxia up-regulated 1** | **-0.15** | **6.6x10^-3^** |
| **ME2** | **malic enzyme 2** | **-0.09** | **6.9x10^-3^** |
| **CGNL1** | **cingulin like 1** | **0.21** | **7x10^-3^** |
| **DIABLO** | **diablo IAP-binding mitochondrial protein** | **-0.10** | **7x10^-3^** |
| **NLE1** | **notchless homolog 1** | **-0.20** | **7x10^-3^** |
| **ERF** | **ETS2 repressor factor** | **-0.13** | **7.1x10^-3^** |
| **PARP6** | **poly(ADP-ribose) polymerase family member 6** | **0.16** | **7.3x10^-3^** |
| **CTNND1** | **catenin delta 1** | **0.19** | **7.7x10^-3^** |
| **POLR1D** | **RNA polymerase I and III subunit D** | **-0.09** | **7.7x10^-3^** |
| **CNOT2** | **CCR4-NOT transcription complex subunit 2** | **-0.09** | **7.8x10^-3^** |
| **SLC30A1** | **solute carrier family 30 member 1** | **-0.13** | **7.8x10^-3^** |
| **IL23R** | **interleukin 23 receptor** | **0.24** | **7.8x10^-3^** |
| **TMEM145** | **transmembrane protein 145** | **0.17** | **7.9x10^-3^** |
| **ZNF485** | **zinc finger protein 485** | **0.15** | **8x10^-3^** |
| **IL1B** | **interleukin 1 beta** | **0.28** | **8x10^-3^** |
| **WDR12** | **WD repeat domain 12** | **-0.07** | **8x10^-3^** |
| **PNPLA8** | **patatin like phospholipase domain containing 8** | **0.22** | **8.1x10^-3^** |
| **UQCC1** | **ubiquinol-cytochrome c reductase complex assembly factor 1** | **-0.11** | **8.2x10^-3^** |
| **CLUAP1** | **clusterin associated protein 1** | **0.14** | **8.2x10^-3^** |
| **FCGRT** | **Fc fragment of IgG receptor and transporter** | **0.17** | **8.2x10^-3^** |
| **IFI27L1** | **interferon alpha inducible protein 27 like 1** | **-0.20** | **8.4x10^-3^** |
| **HSD17B11** | **hydroxysteroid 17-beta dehydrogenase 11** | **-0.18** | **8.6x10^-3^** |
| **SLC35G2** | **solute carrier family 35 member G2** | **0.23** | **8.8x10^-3^** |
| **GCH1** | **GTP cyclohydrolase 1** | **-0.23** | **8.9x10^-3^** |
| **ZNF844** | **zinc finger protein 844** | **-0.17** | **8.9x10^-3^** |
| **ESR2** | **estrogen receptor 2** | **0.18** | **9x10^-3^** |
| **TTC30A** | **tetratricopeptide repeat domain 30A** | **0.15** | **9.2x10^-3^** |
| **ASNA1** | **arsA arsenite transporter, ATP-binding, homolog 1 (bacterial)** | **-0.28** | **9.4x10^-3^** |
| **BCL11A** | **B cell CLL/lymphoma 11A** | **-0.22** | **9.4x10^-3^** |
| **NFATC2IP** | **nuclear factor of activated T cells 2 interacting protein** | **-0.23** | **9.6x10^-3^** |
| **SLC48A1** | **solute carrier family 48 member 1** | **0.17** | **9.6x10^-3^** |
| **CLYBL** | **citrate lyase beta like** | **-0.16** | **9.7x10^-3^** |
| **NUP93** | **nucleoporin 93** | **-0.10** | **9.9x10^-3^** |

*List includes those genes with an uncorrected p-value of < 0.01. PDR – subject with proliferative diabetic retinopathy. nDR – subject with diabetes but no retinopathy.

**Supplementary file 1c. Demographic features of UK Biobank subjects with diabetes used in the diabetic retinopathy GWAS**

|  | **Cases (n=2,332)** | **Controls (n=14,680)** |
| --- | --- | --- |
| **Age years** | **60 (6.86)** | **60 (7.01)** |
| **HbA1c %** | **7.4 (3.5)** | **6.8 (3.4)** |
| **Female %** | **34.70** | **38.50** |
| **T1D (n)** | **8% (187)** | **3.30% (484)** |
| **T2D (n)** | **76.70% (1789)** | **67.90% (9968)** |
| **Unspecified (n)** | **15.3% (356)** | **28.8% (4228)** |

Notes:

Age and HbA1c at enrollment given with mean and (standard deviation).

T1D: Type 1 diabetes (data defined as coded in ICD10 as E10)

T2D: Type 2 diabetes (data defined as coded in ICD10 as E11)

Duration of diabetes is not available.

Case subjects were defined as those who answered “yes” to questionnaire data eyesight field 6148 ‘Diabetes related eye disease’. Control subjects were defined as those who answered “yes” to data field 2443 ‘Diabetes diagnosed by doctor, excluding case subjects.

**Supplementary file 1d. Demographic features of individuals without diabetes from the Coriell Institute for Medical Research NIGMS Human Genetic Cell Repository**

| **Subject** | **Ethnicity** | **Gender** | **Age*(years)** | **BMI** | **Notes** |
| --- | --- | --- | --- | --- | --- |
| **GM14581** | **Caucasian** | **Male** | **18** | **24** |  |
| **GM14569** | **Caucasian** | **Male** | **24** | **N/A** |  |
| **GM14381** | **Caucasian** | **Female** | **20** | **21** | **#** |
| **GM07012** | **Caucasian** | **Female** | **N/A** | **N/A** | **CEPH^%^** |
| **GM14520** | **Caucasian** | **Female** | **22** | **32** |  |
| **GM11985** | **Caucasian** | **Female** | **N/A** | **N/A** | **CEPH^%^** |
| **GM07344** | **Caucasian** | **Female** | **N/A** | **N/A** | **CEPH^%^** |

Notes:

* At time of sampling

# Family history of diabetes

% Repository Linkage Families

N/A Not Available
