# Supplementary material for: Integration of genomics and transcriptomics predicts diabetic retinopathy susceptibility genes
Source: eLife. 2020 Nov 9;9:e59980. doi: 10.7554/eLife.59980 (PMC7728435; doi:10.7554/eLife.59980)
Supplement: Transparent reporting form [file elife-59980-transrepform.docx]

***eLife’s* transparent reporting form**

We encourage authors to provide detailed information *within their submission* to facilitate the interpretation and replication of experiments. Authors can upload supporting documentation to indicate the use of appropriate reporting guidelines for health-related research (see [EQUATOR Network](http://www.equator-network.org/%20)), life science research (see the [BioSharing Information Resource](https://biosharing.org/)), or the [ARRIVE guidelines](http://www.plosbiology.org/article/info:doi/10.1371/journal.pbio.1000412) for reporting work involving animal research. Where applicable, authors should refer to any relevant reporting standards documents in this form.

If you have any questions, please consult our Journal Policies and/or contact us: [editorial@elifesciences.org](mailto:editorial@elifesciences.org).

**Sample-size estimation**

- You should state whether an appropriate sample size was computed when the study was being designed
- You should state the statistical method of sample size computation and any required assumptions
- If no explicit power analysis was used, you should describe how you decided what sample (replicate) size (number) to use

This information can be found in the Methods section under:

1. Gene Expression Analysis

Sample size estimation was based on our prior published work in assessing lymphoblastoid cell line (LCL) response to glucose [1]. Specifically, we found high glucose induced upregulation of gene expression in LCLs. We measured a panel of genes previously implicated in diabetic retinopathy and leukocyte associated inflammation. Fold change response to glucose was determined for each gene. All six genes demonstrated greater than mean 2x FC induction in high glucose (2.05-2.82). The control GAPDH gene did not show any change with high glucose. Based on these findings, we generated a power curve using the difference between two independent means taken from each of the two groups. With an effect size of 2 there is 95% power in a sample size of 15 individuals. Hence, we felt the study was sufficiently powered to identify at a minimum those genes that showed two-fold or greater gene expression difference between two groups. Moreover, the gene expression portion of this study design was purely hypothesis generating and used for discovery purposes. Validation was achieved through genetic association, a completely orthogonal approach.

1.	Grassi, M.A., et al., *Lymphoblastoid Cell Lines as a Tool to Study Inter-Individual Differences in the Response to Glucose.* PLoS One, 2016. **11**(8): p. e0160504.

Please outline where this information can be found within the submission (e.g., sections or figure legends), or explain why this information doesn’t apply to your submission:

**Replicates**

- You should report how often each experiment was performed
- You should include a definition of biological versus technical replication
- The data obtained should be provided and sufficient information should be provided to indicate the number of independent biological and/or technical replicates
- If you encountered any outliers, you should describe how these were handled
- Criteria for exclusion/inclusion of data should be clearly stated
- High-throughput sequence data should be uploaded before submission, with a private link for reviewers provided (these are available from both GEO and ArrayExpress)

Please outline where this information can be found within the submission (e.g., sections or figure legends), or explain why this information doesn’t apply to your submission:

Methods section, under:

1. Quality Control for Gene Expression
2. Gene expression profiling: biologic replicates
3. Gene Expression Analysis
4. Relative EBV Copy number
5. Growth Rate Measurement
6. Data and Code Availability
7. Figure 5 legend.

**Statistical reporting**

- Statistical analysis methods should be described and justified
- Raw data should be presented in figures whenever informative to do so (typically when N per group is less than 10)
- For each experiment, you should identify the statistical tests used, exact values of N, definitions of center, methods of multiple test correction, and dispersion and precision measures (e.g., mean, median, SD, SEM, confidence intervals; and, for the major substantive results, a measure of effect size (e.g., Pearson's r, Cohen's d)
- Report exact p-values wherever possible alongside the summary statistics and 95% confidence intervals. These should be reported for all key questions and not only when the p-value is less than 0.05.

Please outline where this information can be found within the submission (e.g., sections or figure legends), or explain why this information doesn’t apply to your submission:

Methods section, under:

1. Quality control for gene expression
2. Gene expression analysis
3. Gene set enrichment analysis (GSEA)
4. Expression quantitative trait loci (eQTL)
5. Genome wide association study (GWAS)
6. Mendelian Randomization

(For large datasets, or papers with a very large number of statistical tests, you may upload a single table file with tests, Ns, etc., with reference to sections in the manuscript.)

**Group allocation**

- Indicate how samples were allocated into experimental groups (in the case of clinical studies, please specify allocation to treatment method); if randomization was used, please also state if restricted randomization was applied
- Indicate if masking was used during group allocation, data collection and/or data analysis

Please outline where this information can be found within the submission (e.g., sections or figure legends), or explain why this information doesn’t apply to your submission:

Methods section, under:

1. Cell lines
2. Genome wide association study (GWAS)

**Additional data files (“source data”)**

- We encourage you to upload relevant additional data files, such as numerical data that are represented as a graph in a figure, or as a summary table
- Where provided, these should be in the most useful format, and they can be uploaded as “Source data” files linked to a main figure or table
- Include model definition files including the full list of parameters used
- Include code used for data analysis (e.g., R, MatLab)
- Avoid stating that data files are “available upon request”

1. We have provided source data and source code for all the figures and tables, except for figure 5a and 5b (drawings), figure 3a (flowchart) and figure 4- figure supplement 1 (histopathology findings). We have also included links and references where appropriate.
2. Figure 3 additional source files are available on Dryad at <https://doi.org/10.5061/dryad.zkh18938j>
3. Additional data files can be found here: microarray expression data at Gene Expression Omnibus (GEO) under accession code GSE146615 and diabetic retinopathy GWAS data at UKBB archive (<https://oxfile.ox.ac.uk/oxfile/work/extBox?id=825146B4380F72048D>).

Please indicate the figures or tables for which source data files have been provided:
